# Temperature Compensation Method for Raster Projectors Used in 3D Structured Light Scanners

**DOI:** 10.3390/s20205778

**Published:** 2020-10-12

**Authors:** Marcin Adamczyk

**Affiliations:** Institute of Micromechanics and Photonics, Faculty of Mechatronics, Warsaw University of Technology, ul. Św. Andrzeja Boboli 8, 02-525 Warsaw, Poland; marcin.adamczyk@pw.edu.pl; Tel.: +48-22-234-86-02

**Keywords:** temperature effect, 3D imaging, raster projector, 3D structured-light scanner, temperature compensation

## Abstract

Raster projectors are commonly used in many various measurement applications where active lighting is required, such as in three-dimensional structured light scanners. The effect of temperature on the raster projector, in some conditions, can lead to significant deterioration of the measurements performed with such a scanner. In this paper, the outcomes of several experiments concerning the effects of temperature on raster projectors are presented. The described research is focused on the thermal deformations of projected images caused by common thermal effects observed in projectors: those caused by the warming-up process and changes in ambient environmental temperature. A software compensation method is also presented. It is suitable for implementation in any existing measurement method that uses raster projectors. The results of performed verification experiments show that the developed compensation method can decrease the thermal drift of the projected images by up to 14 times in the ambient temperature range 14–42 °C.

## 1. Introduction

Three-dimensional (3D) structured light (SL) scanners are currently used in many scientific, industrial and engineering fields. In order to meet the high requirements of the modern market, SL scanners need to be better, faster, and more accurate [1,2,3,4,5,6,7]. Consumers require scanners that carry out metrologically correct measurements. Their measurement uncertainty can be described by a dedicated parameter [3,6,8,9,10,11,12,13] called the Maximum Permissible Error ($E_{MPE}$) [6,9,10,11,14]; methods for its calculation have been described in the ISO 10360-8 [14] standard and the VDI/VDE2634 [10] recommendations. The error $E_{MPE\;}\;$can be determined using dedicated validation artefacts. Scanner calibration and its metrological validation (which involves the estimation of $E_{MPE}$) is performed in a laboratory using a stabilized environment (i.e., constant temperature, low and constant humidity, no vibrations, and no external light sources) [3,6,9,10,12,13,14,15]. Further measurements are often performed outside the laboratory, where the environmental conditions can vary. In particular, the temperature is one of the most critical factors that can cause significant measurement errors [3,6,13,15]. Some research has shown that the temperature range in which a 3D SL scanner maintains its metrological properties can be relatively small, up to 5 deg [15,16,17]. The effects of temperature on a 3D SL scanner can be observed in three main fields: the effect of temperature on the field detector (most often a digital camera with lens), the effect of temperature on the mechanical base of the scanner, and the effect of the temperature on the raster projectors.

Some papers have described the effects of temperature on the digital cameras used in 3D SL scanners. Handel, in [18,19,20], described a method for the compensation of temperature on the image drift of the camera. Handel proposed a linear model that can be used for compensation of the effect of temperature on the intrinsic parameters of the camera. Podbreznik and Potočnik described similar studies in [21]. Extensive studies in this field and another compensation method were described in [22]. The influence of temperature changes on the geometric stability of smartphone and cheap Raspberry Pi cameras was described in [23], in which the authors proposed a compensation method. In [24], I also proposed a compensation method that can be used in any camera calibration model (i.e., not only a pinhole camera model).

When studying the effect of temperature on the uncertainty of the 3D SL scanner, the effect of the temperature on digital cameras is not the only factor that needs to be considered. The effects of temperature on another two 3D SL scanner units—the mechanical base and the raster projector—also need to be investigated. The compensation method of the effect of temperature on the scanner base was the scope of my previous study, described in [25]. At the same time, no papers have described the impact of temperature on the thermal image drift and the deformation of images displayed by the raster projector. There are available papers that have described the effect of temperature on the ageing process of a projection matrix [26,27,28,29]. There also can be found papers that have described the effect of temperature on the intensity of projected images, color reproduction, color temperature or signal-to-noise ratio [28,29,30,31]. The point is that there is no available literature that reports the effect of temperature on the image drift and deformation of the images projected by raster projectors.

**Figure 1:**
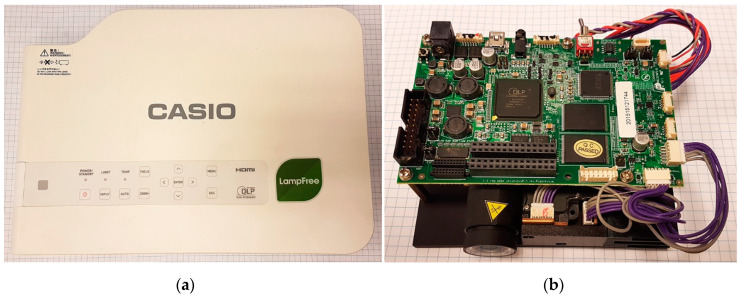
The projectors used in the experiments: (**a**) business-class projector DLP Casio XJ-A252; and (**b**) development kit projector DLPLightCrafter 4500.

In the construction of 3D SL scanners, commercial, business-class projectors are often used (Figure 1a) [1,5,15,32,33,34,35,36,37]. This is due to their relatively low price, high image quality, and easy integration with other scanner components. Business projectors are usually equipped with an HDMI, DisplayPort, or VGA input, which allows for their easy integration with a computer and scanner control software. The drawback of using commercial business projectors is the fact that their design has not been optimized for use in 3D scanners [8,15]. Consequently, their lenses and the optical path are often not rigid enough. Some projectors have a cooling system that vibrates the optical system. These vibrations do not affect the perception of the image when using the projector for standard usage but, when using the projector as a source of projected images in a 3D scanner, such vibrations are unacceptable. Another disadvantage is additional hardware improvements which, in 3D scanners, may lead to deterioration of the quality of scans or scanner de-calibration. These are all kinds of image correction functions (e.g., keystone correction or resolution interpolation), as well as functions related to the adaptive change in image intensity depending on external light. These types of functions can affect the intensity, contrast, and linearity of projected images. An additional feature, which is not always desired in the projectors used in 3D scanners, is the fact that color images are displayed. In 3D SL scanners, raster images are most often monochrome [2,3,8,38,39,40]. The use of all three projection channels may cause deterioration of the quality of the designed rasters, due to the chromatic aberrations between the R, G, and B channels. For this reason, often, only one color channel is used to project fringe images.

Development kit projectors are an alternative to business projectors; for example, the Texas Instruments Digital Light Processing (DLP) LightCrafter family projectors (Figure 1b) [41,42,43,44,45]. These are projectors equipped with a DLP system and a Digital Micromirror Device (DMD) matrix. They allow for the projection of fringe images to be directly implemented in the memory (i.e., an external signal source is not required for image projection), which allows for quick measurements by significantly facilitating the synchronization of the display with image registration by the camera. The projector has no housing; it is delivered as a so-called evaluation board, on which the user can test various hardware and software solutions. The advantage of this projector is that it does not come with any of the previously mentioned features dedicated to business projectors.

Business projectors and development-kit projectors have one more important feature that should be taken into account when selecting a projection unit for a 3D SL scanner: the effect of temperature changes on the displayed image is not compensated for.

The compensation of thermal effect on projectors is a crucial issue when talking about 3D SL scanners equipped with one camera. When 3D SL scanner is equipped with at least two (or more) cameras, then there is a possibility to recalibrate the phase values for every single measurement, which allows for the “natural” compensation of the effect of temperature on the projector. It requires a more complicated approach to the measurement sequence, and not every multi-camera scanner is equipped with this feature. The condition that must be fulfilled is the stability of the camera calibration and the stability of mutual calibration between cameras. However, there are still available 3D scanners that are equipped with only one camera (HP 3D Structured Light Scanner Pro S3 [46], Smarttech3D [47]), where the phase values are calibrated during the calibration process.

This paper presents the outcome of research in the field of thermal effect observed in digital projectors; also, the temperature compensation method is proposed. It can significantly decrease the thermal drift and deformation of the projected image. This paper is also the continuation of the studies described in [15,24,25], where the effects of temperature on the digital cameras, mechanical base, and the whole 3D SL scanner were introduced.

The following section (Section 2) describes the test stand setup used in the experiments. In Section 3, the results of preliminary studies and the thermal image drift caused by the warming-up processes of two commonly used in SL scanner projectors—DLP Casio XJ-A252 (Figure 1a) [48] and DLP LightCrafter 4500 Figure 1b [41] are described. In Section 4, the outcomes of studies using varying ambient temperatures on the DLP LightCrafter 4500 projector are presented, and the compensation model is introduced. The results of the experiment and from applying the compensation model are provided in Section 4.4**.**

## 2. The Test Stand

**Figure 2:**
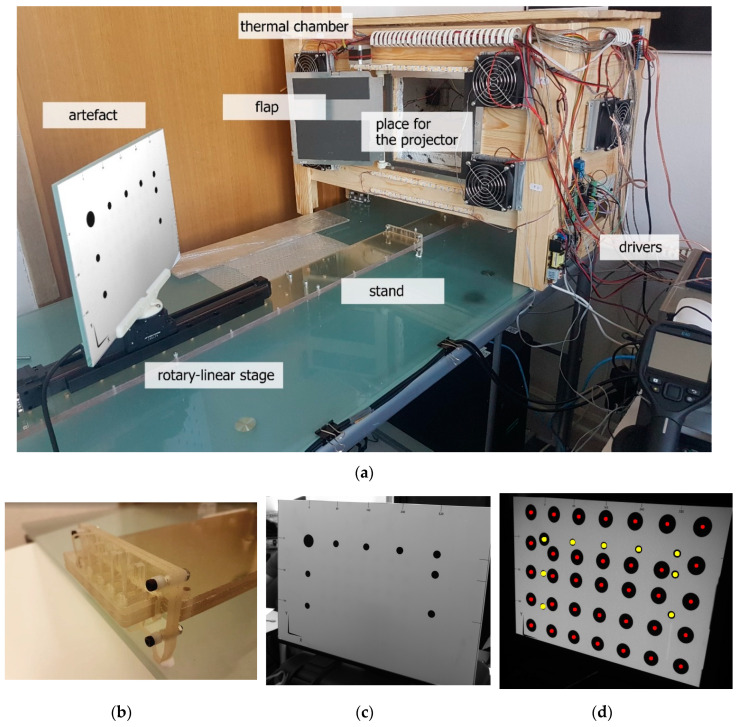
Test stand and the validation artefact: (**a**) overview of the test stand, with the Robax stand and the thermal chamber: (**b**) the athermalized design of the stand—the Robax keys preventing the thermal deformation of the glued connection of the stand; (**c**) the validation artefact with the matrix of printed markers; and (**d**) the same validation matrix (markers printed on the artefact are marked with yellow dots) with the projected matrix of projector-related markers (marked with red dots).

A dedicated test stand was designed to conduct experiments with varying ambient temperatures, analyzing the consequent effect on the raster projector (Figure 2a). The test stand was designed as follows: the projector and the validation artefact (which was also a projection screen) were mounted on a dedicated stand made of Robax glass (with a coefficient of thermal expansion α20−700℃ =(0±0.5)×10−6[1K]) [49,50]. The stand was athermalized by specialized keys made from Robax, which prevented the glued connections from deforming due to varying temperature (Figure 2b). One side of the stand (where the projector was mounted) was hidden inside the thermal chamber. The validation artefact was placed in front of the projector. It was mounted on the rotary-translation stage and placed outside the thermal chamber. An additional camera unit (PtGrey Grasshopper 2.0 GS2-GE-50S5M-C [51]) was used to register the frames projected by the projector onto the artefact. The thermal chamber was equipped with an automatic flap, which was opened only during the time needed to capture the image with the additional camera.

The whole test stand (including the additional camera unit) was placed in the laboratory, in which an air conditioning system controlled the temperature. The test stand was designed in such a way that the varying ambient temperature acted only on the projector unit; that is, the validation artefact and the additional camera were not exposed to temperature changes. The validation artefact was composed of a flat glass plate with a pattern of black markers on a white surface printed onto it (Figure 2c). In order to detect the thermal image drift caused by the temperature changes, the projector projected an image with a matrix of markers onto the validation artefact (Figure 2d). Such a solution allowed for detecting and quantitively estimating of the image drift of the projected image and for checking whether the positioning between the camera, projector, and validation artefact remained the same during the experiment. The test stand was also equipped with a FLIR E40 thermal camera [52] and a temperature registration unit MultiCon CMC by Simex Ltd. [53] with a set of Pt100 sensors (class A, four wires).

## 3. Preliminary Studies

In the preliminary studies, the warming-up processes of the two projectors were investigated. These studies were focused on determining the time that was needed to achieve thermal equilibrium, and the maximum observed temperature on the projector surface and the effect of projector warming-up on the deformation of the projected image. Therefore, two kinds of experiments were conducted:

- The first experiment was related to registration of the temperature and time to reach the projector’s thermal equilibrium; and
- The scope of the second experiment was to register the thermal drift and the deformation of the projected image.

### 3.1. The Warming-Up Process—Thermal Equilibrium

This experiment was conducted twice: in the first test, the projector displayed a uniform white frame (all pixels were set to 255 intensity level) and, in the second one, each projector displayed a uniform black frame (all pixels were set to 0 intensity level). This test was conducted on the test stand introduced in the Section 2, but the thermal chamber was turned off, and the automatic flap was left open. The validation artefact was not used in this experiment.

**Figure 3:**
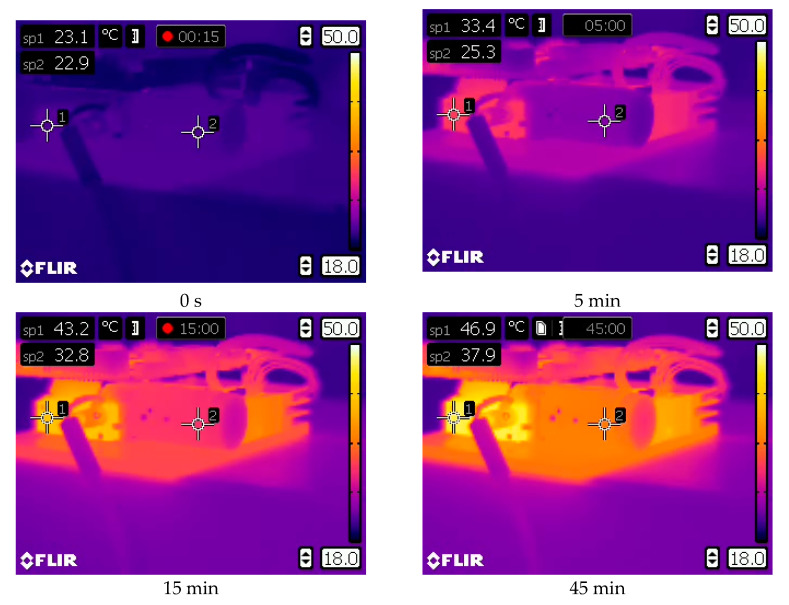

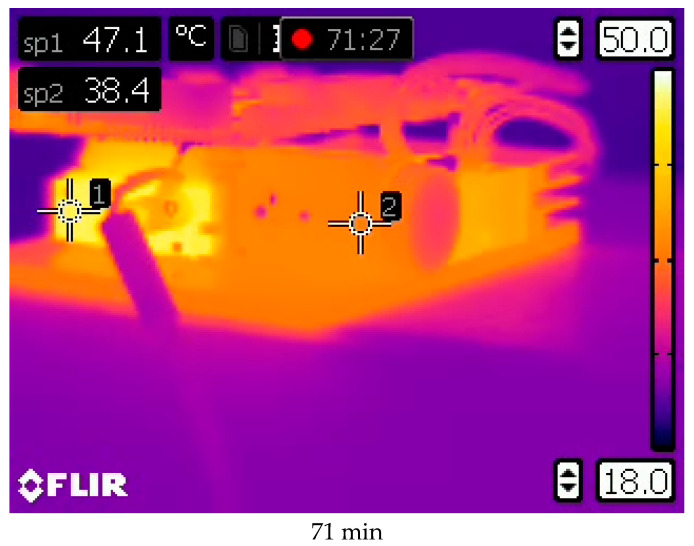
The warming-up process of the DLP LightCrafter 4500 projector, registered with thermal camera Flir E40. The colors on the presented images show the projector temperature in the first 71 min after turning on the power supply. The LED source is the warmer place on the projector (measuring point sp1).

**Figure 4:**
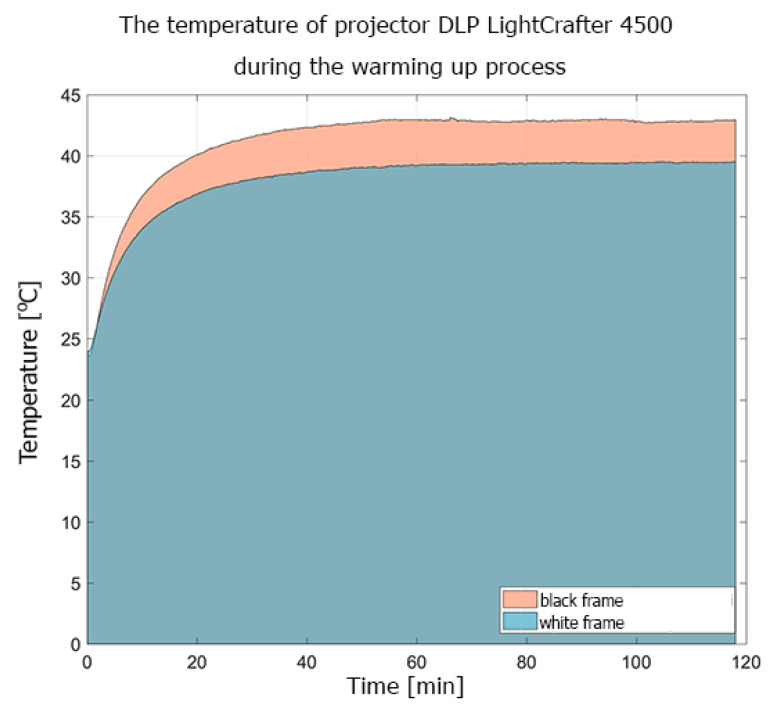
The temperature registered during the warming-up experiment of the DLP LightCrafter 4500 projector displaying white and black frames. The temperature was measured by the PT100 sensor attached to the LED light sources.

Figure 3 shows the warming-up process of the DLP LightCrafter 4500 projector, as registered by the thermal camera Flir E40. The experiment lasted about 120 min (only the first 71 min are shown on the thermal camera). In Figure 3, the temperature sensor PT100 can also be seen, attached near the LED light sources (near the measuring point sp1). The temperatures registered by this sensor during the two experiments (i.e., displaying white and black frames) are presented in Figure 4.

**Figure 5:**
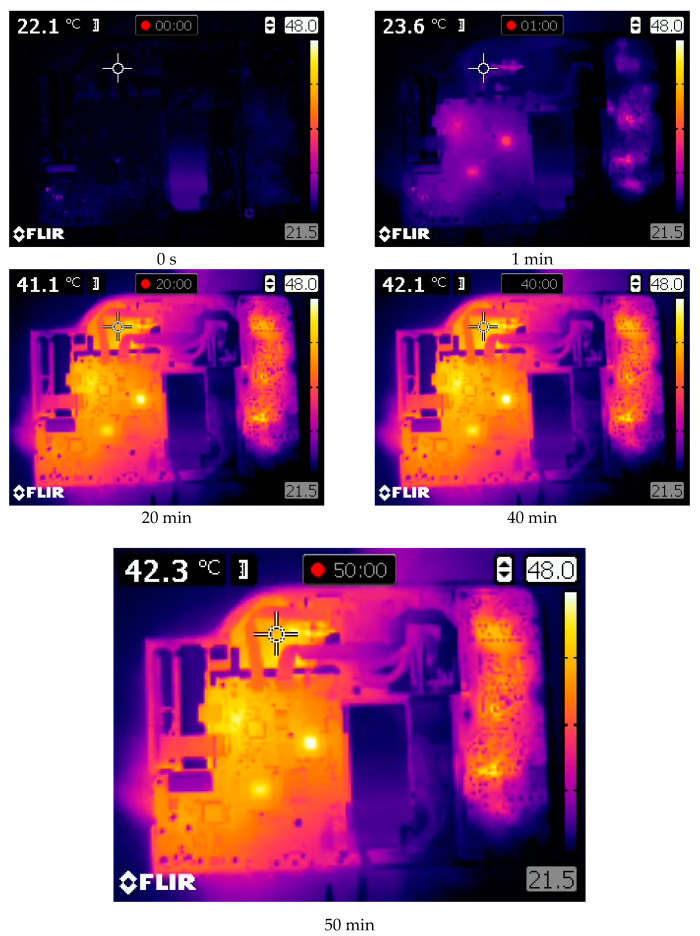
The warming-up process of the DLP Casio XJ-A252 projector, registered with the thermal camera Flir E40. The top plate of the projector was removed to show the inside of the projector. The measuring point is located on the housing of the optical system.

In Figure 5, the warming-up process of the projector DLP Casio XJ-A252, registered by the thermal camera Flir E40, is shown. The experiment lasted about 50 min. One measurement point was defined, placed near the optical system. For this experiment, the top plate of the projector housing was removed.

**Figure 6:**
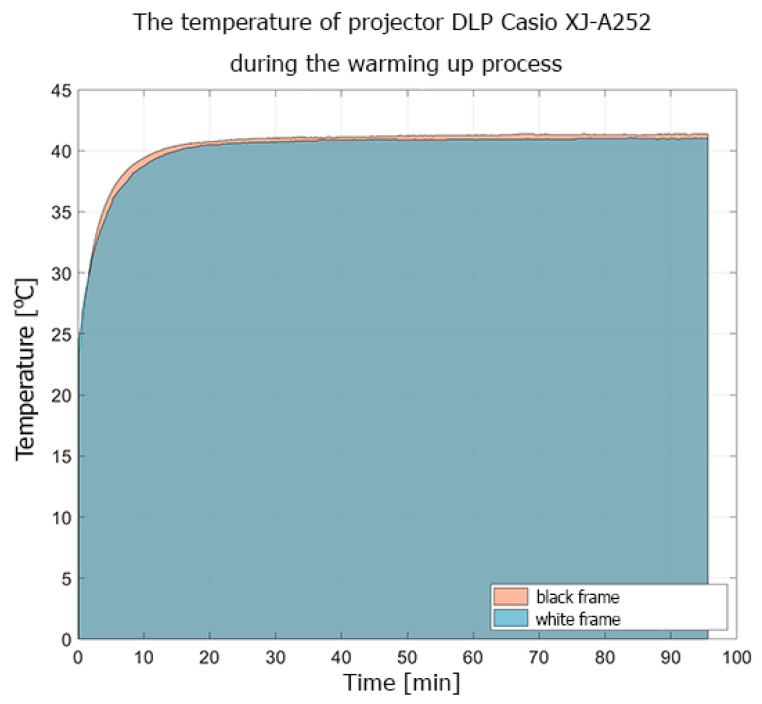
The temperature registered during the warming-up experiment of the DLP Casio XJ-A252 projector displaying white and black frames. The temperature was measured by the PT100 sensor attached to the optical system housing.

The conducted experiments show that the heat distribution on the surface of the projectors was not uniform. The LightCrafter 4500 reached its thermal equilibrium about 50–55 min after power-up, while that for the Casio XL-A252 projector was after about 20 min. The difference in the registered temperature while displaying black and white frames was caused by the fact that, during the projection of the white frame, all micro-mirrors from the DMD sensors reflected light to the projection lens but, during the black frame projection, all micro-mirrors reflected light to the absorber. For the DLP LightCrafter 4500, the difference in temperature during the projection of black and white frames reached up to 5–6 ℃. In the Casio projector, the difference was not as significant, about 1 ℃ (Figure 6). This difference in projector behavior was caused by the adaptive cooling system implemented in the Casio XJ-A252 projector.

The most important conclusion from these experiments is that the temperature of the projector may be related to the displayed image. This could be crucial information when considering the usage of a particular projector in the 3D SL scanner. When conducting measurements with a 3D SL scanner, displaying a sequence of measurement images takes up only a certain period of the entire scanning cycle [1,2,3,8,35,36]. Depending on the detector used and the number of displayed frames, the projection time usually ranges from a fraction of a second up to several dozen seconds. Outside of these periods, the projector in the scanner can project any image and, in practice, full black frame projection is often used. The conducted tests showed that, when the black frame was displayed, the temperature of the projector may rise by several degrees due to the light beam being directed into the absorber.

### 3.2. The Warming-Up Process—Thermal Drift and Deformation of the Projected Image

The scope of this experiment was to register the thermal drift and deformation of the projected image. The thermal chamber was turned off, and the automatic flat was left open, but the validation artefact and the external camera were used to capture the projected images. Immediately after turning on the projector and projection of the prepared image, the external camera began to register the frames.

**Figure 7:**
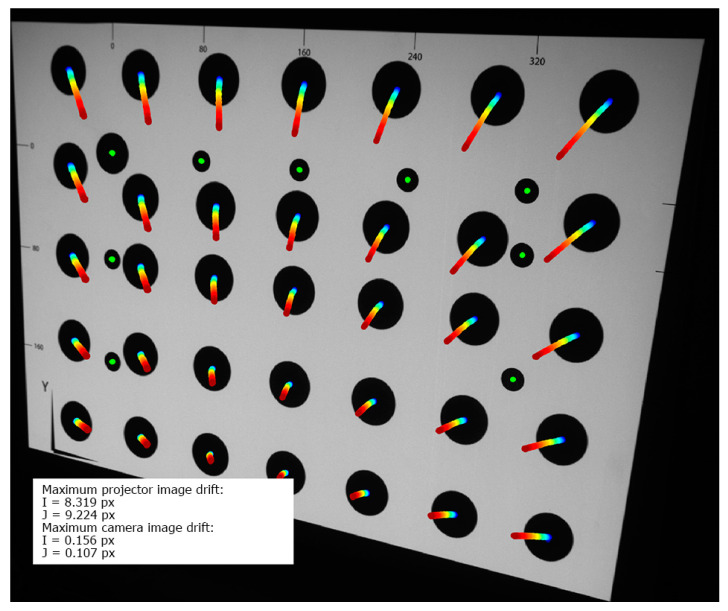
Thermal image drift of the LightCrafter 4500 during the warming-up process. The background frame is the first registered frame (immediately after turning on the projector). There are two types of markers: thirty-five big black markers, displayed by the projector, and nine smaller black markers (marked with bright green color), printed on the validation artefact. The marker trajectories are multiplied 20 times. The colors show the temperature of the projector (blue dot: temperature around 24 ℃, red dot: temperature around 43 ℃).

In Figure 7, the thermal image drift caused by the warming-up process of the LightCrafter4500 projector is shown. The first registered frame is the background of this figure (it was captured immediately after the projector was turned on at the beginning of the warming-up process). There are two types of markers visible: thirty-five large black markers (marked with their trajectories), which were projected by the projector, and nine smaller black markers (marked with bright green color), which were printed on the validation artefact. In order to obtain the trajectory of projected markers, their coordinates are extracted from each captured frame and marked on the background image with the colored indicator (the color denotes the temperature of the projector). The shift of each marker canter is multiplied 20 times. The image drift presented Figure 7 shows that the projected image had shrunk around the center-bottom point of the image. The maximum drift of the projected markers was equal to $I_{max}=$ 8.319 px horizontal and $J_{max}=$ 9.224 vertical, which is equal to 1.89 mm horizontal shift and 2.1 mm vertical shift in the artefact plane. The markers printed on the validation artefact remained stable during the whole experiment. Its maximum drift was no higher than 0.156 px, which means that the positioning between the external camera and the validation artefact remained stable during the whole experiment and that image drift and deformation were caused only due to thermal effects in the projector.

**Figure 8:**
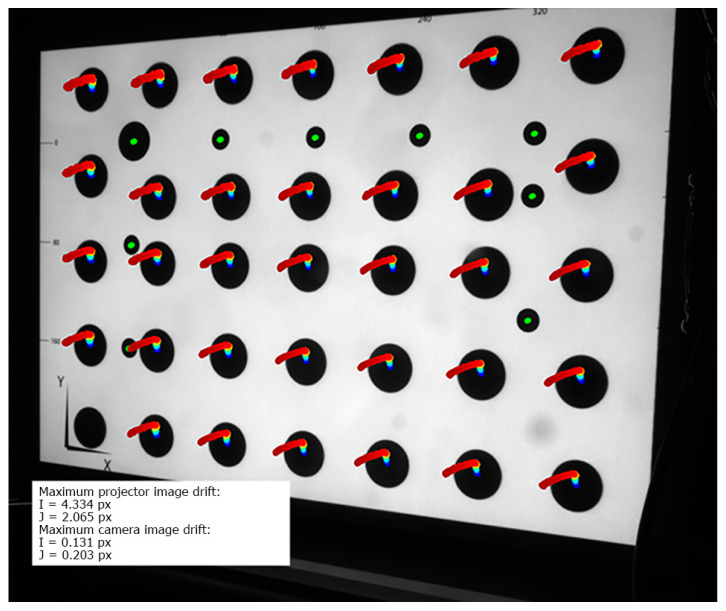
Thermal image drift of the DLP Casio XJ-A252 during the warming-up process. The marker trajectories are multiplied 20 times. The colors show the temperature of the projector (blue dot: temperature around 24 °C, red dot: temperature around 41 °C).

**Figure 9:**
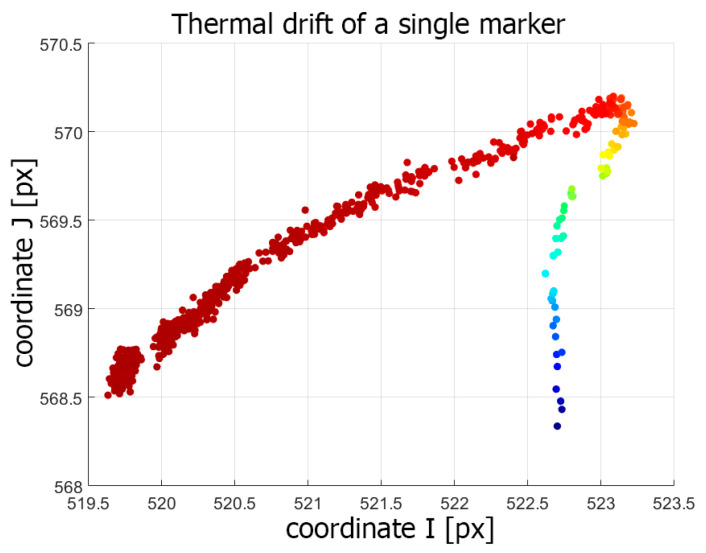
The trajectory of a single marker during the warming-up process of the Casio XJ-A252 projector.

In Figure 8, the thermal image drift of the Casio XJ-A252 projector is presented. All markings and the test method were the same as for the LightCrafter 4500 warming-up experiment. The maximum drift of the projected markers was equal to $I_{max}=\;$4.334 px horizontal and $J_{max}=$ 2.065 vertical, which is equal to 1.05 mm horizontal shift and 0.51 mm vertical shift in the artefact plane. The markers printed on the validation artefact remained stable during the whole experiment, with maximum drift no higher than 0.203 px. All marker trajectories were similar. In Figure 9, the trajectory of a single marker is shown. In the first stage of the warming-up process, a global shift in the vertical direction can be observed. After a while of warming-up (at around 37 ℃), the trajectories turned to the left. This type of temperature drift was most likely due to the uneven heating of the projector. During the initial warming-up phase, the nature of the thermal drift was caused by some particular part of the projector. When the heat generated by the projector began to flow to other parts of the projector, they also began to deform, and the nature of the drift changed. Another conclusion from the analysis of Figure 9 is that the drift progressed, even though the projector temperature did not change. The reason for this phenomenon was the heterogeneous temperature distribution on the projector’s surface and that the external temperature sensor was located in a place where the projector locally reached its thermodynamic equilibrium much faster. However, this state was not the same as the state of thermodynamic equilibrium of the entire projector. Thus, in the approach to building a compensation model for this projector model, one should choose a different place to measure the temperature.

## 4. Compensation

### 4.1. Tests in the Thermal Chamber

In order to determine the effect of external temperature changes on the projector, only the LightCrafter 4500 projector model was used, as it was the most representative and most suitable for usage in a 3D SL scanner. The experiments were conducted on the test stand described in Section 2. The chamber inspection flap remained closed while the set temperature inside the chamber was established. At the beginning of the test, the projector and external camera were turned on and left for about 120 min, in order to achieve a stable temperature. At this temperature (called the reference temperature), the external camera collected a series of reference frames. Then, the temperature inside the chamber was changed and left for at least 3 h for the temperature of the projector and the chamber to stabilize again. For each stabilized temperature, the inspection flap of the chamber was opened for a very short time, only necessary for the external camera to capture images of the artefact illuminated by the projector. The projector was positioned in such a way that it projected through the inspection flap. This setup allowed the temperature of the projector to be maintained constant (i.e., the time needed for the acquisition of images was so short that it did not affect the temperature of the projector) and, at the same time, to isolate the influence of varying temperature only on the projector. The temperatures of the validation artefact, external camera, and other elements of the test stand were not affected, being outside the chamber. The entire experiment lasted about 63 (continuously) h.

**Figure 10:**
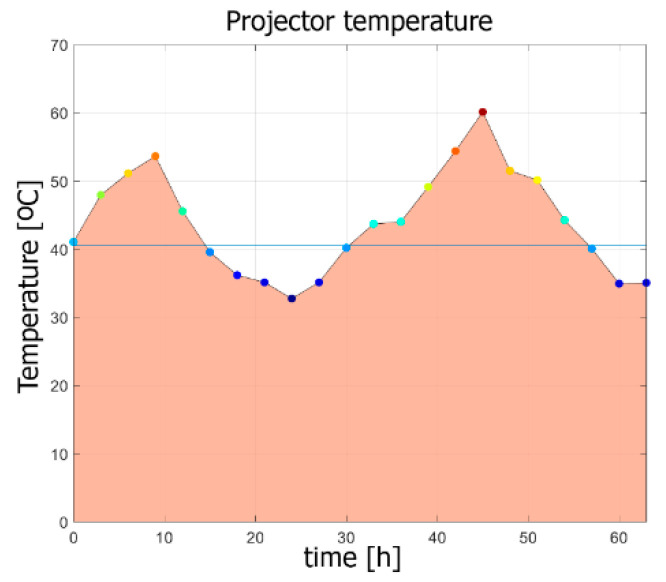
Projector temperature during the tests in the thermal chamber. The horizontal blue line indicates the reference temperature $T_{ref}=42\;℃$.

Figure 10 presents the projector temperature during the tests in the thermal chamber. The temperatures shown in the graph represent the actual temperatures of the projector (measured by an external sensor located near light sources) when the external camera was capturing frames to determine the thermal drift of the projected images. The projector reference temperature $T_{ref}=42\;℃$ corresponded to an ambient temperature of 24 ℃ (inside the chamber). The lack of symmetry in the temperature function, as visible in the graph, resulted from the fact that used thermal chamber did not have enough cooling power, in relation to the heating power of the projector. Hence, the lowest temperature of the projector was 32 ℃ (corresponding to 14 ℃ ambient temperature), and the highest was 60 ℃ (42 ℃ ambient).

**Figure 11:**
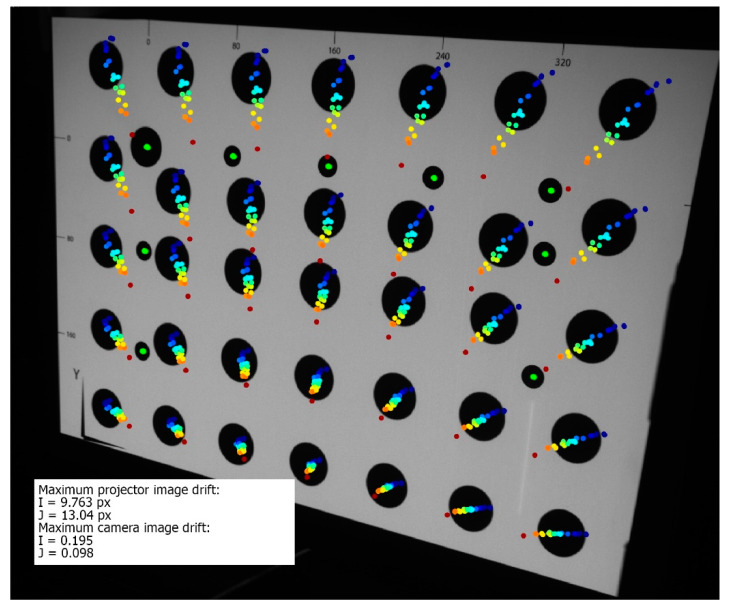
Thermal image drift registered during tests performed in the thermal chamber. The marker trajectories are multiplied 20 times. The marker colors show the temperature of the projector, corresponding to the colors in Figure 10.

**Figure 12:**
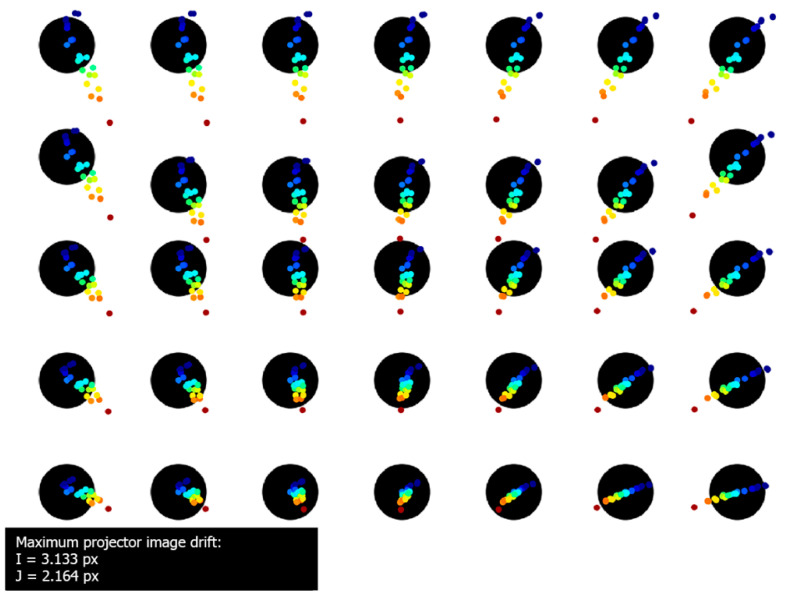
Thermal image drift of the DLP LightCrafter4500 projector, presented in the coordinates of the projector. The marker colors show the temperature of the projector and correspond to the colors in Figure 10.

Figure 11 presents the registered thermal drift of the projected images. The maximum drift of the projected markers was equal to: $I_{max}=9.763\;\mathrm{px}$ horizontal and $J_{max}=13.04\;\mathrm{px}$ vertical, equal to 2.22 mm horizontal shift and 2.96 mm vertical shift in the artefact plane. The markers printed on the validation artefact remained stable during the whole experiment, with maximum drift no higher than 0.195 px. The image thermal drifts presented in Figure 7, Figure 8 and Figure 11 are presented in the coordinates of the external camera. Therefore, the coordinates had to be transformed into the coordinates relative to the displayed image (i.e., the projector coordinates). Polynomial fitting was used to approximate the functions that describe the relationship between projector and camera coordinates. Figure 12 shows the image thermal drift recorded during the experiment, in the coordinates related to the displayed image. The background of the image is the bitmap displayed by the projector, and subsequent shifts of the marker centers are marked with colors (the shifts were scaled 20×). The maximum values of the thermal drift of the image were $I_{max}=3.13\;\mathrm{px}$ horizontal and $J_{max}=2.16\;\mathrm{px}$ vertical.

### 4.2. The Compensation Model

The coordinates of the centers of the markers (in the projector coordinate system) at different temperatures were used to determine the compensation model. The independent variables utilized in the compensation model included the set of calculated marker center coordinates $I_{uncomepnsated}$ and $J_{uncompensated}$ for various projector temperatures. The dependent variables were the calculated centers of the markers $I_{ref}\;$and $J_{ref\;}$for the reference temperature. The compensation model function was an nth degree polynomial, $P^{n}$, which describes the thermal image drift caused by varying the temperature of the projector. Polynomial fitting was used to determine the coefficients associated with the model [54].

**Figure 13:**
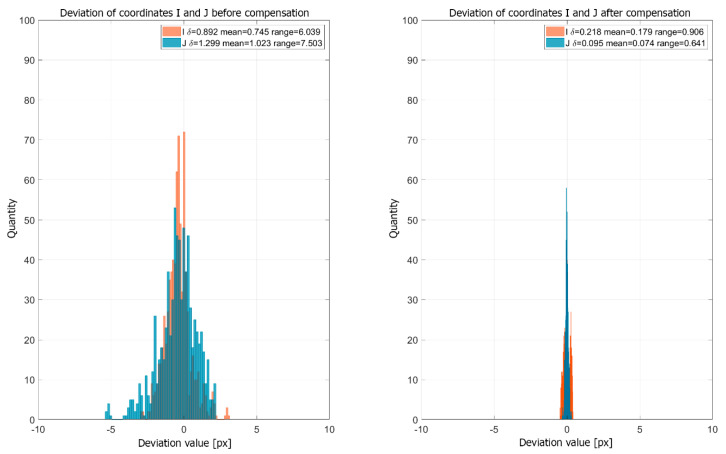
Histogram of deviations of projector marker coordinates before and after the application of the compensation model.

The best fitting results were observed when using a 5th degree polynomial. Figure 13 shows histograms presenting the values of the coordinates of the projector markers at different temperatures relative to the coordinates at the reference temperature before and after compensation. The presented histograms show that, after application of the compensation model, the projector marker coordinate deviations decreased significantly. The mean absolute value before compensation was equal to $I_{mean}=0.892\;\mathrm{px}$ and $J_{mean}=0.299\;\mathrm{px}$ and those after compensation decreased to $I_{mean}=0.218\;\mathrm{px}$ and $J_{mean}=0.095\;\mathrm{px}$. The thermal image drift range before compensation was $I_{range}=6.039\;\mathrm{px}$ and $J_{range}=7.503\;\mathrm{px}$ and that after compensation was $I_{range}=0.906\;\mathrm{px}$ and $J_{range}=0.641\;\mathrm{px}$. The reduction in the deviation of projector marker coordinates confirmed that the calculated compensation model allowed for a reduction in image deformation caused by changes in the external temperature of the projector.

### 4.3. The Verification Experiment

Another validation experiment was performed in order to prove the correctness of the constructed compensation model. Using Matlab [55] and a function that applies geometric transformations to images, the projected bitmap was deformed according to the calculated compensation model. The imwarp [56] function was used where linear interpolation was used to generate TransformedImage. The ImageToTransform was the reference bitmap originally projected by the projector, and DisplacementField was determined using the calculated compensation polynomials.

TransformedImage = imwarp (ImageToTransform, DisplacementField)(1)

**Figure 14:**
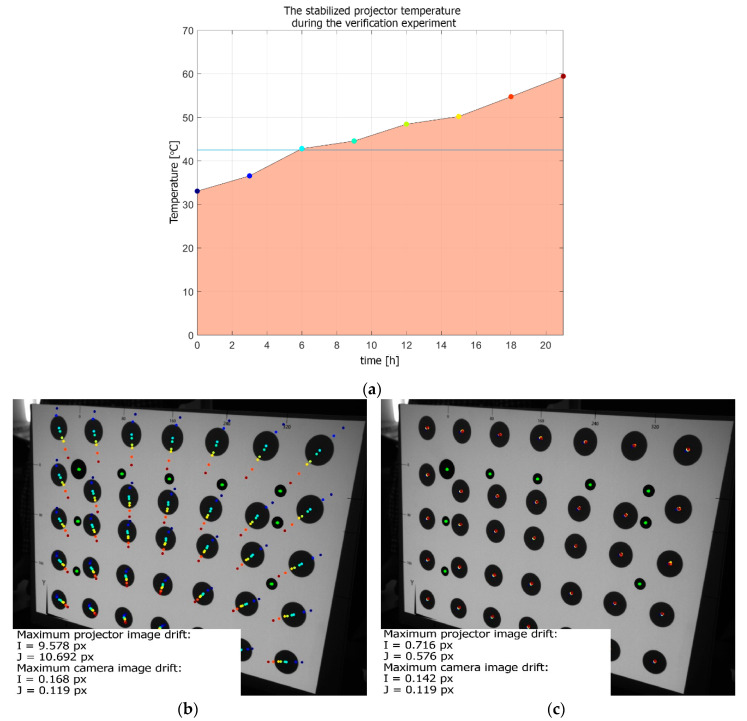
Results of the verification experiment: (**a**) the stabilized projector temperatures; (**b**) the trajectories of the markers calculated for the projection of the undeformed (original) bitmap; and (**c**) the marker trajectories calculated for the projection of the bitmaps deformed using the calculated compensation model.

The transformed images were calculated for the entire temperature range of the projector, from 32 ℃ to 60 ℃ with a step of 0.1 ℃. This resulted in a set of 271 images for the entire temperature range. Another set of images, projected by the projector in varying ambient temperature (Figure 14a), was collected. At each stabilized temperature, two kinds of frames were captured by the external camera: one frame with the projector displaying the original (undeformed) bitmap and another frame with the bitmap transformed by the imwarp function with respect to the corresponding projector temperature. Figure 14b presents the trajectories of the markers calculated for projection using the undeformed (uncompensated) bitmap. The maximum image drift was equal to $I_{max}=9.578\;\mathrm{px}$ horizontal and $J_{max}=10.692\;\mathrm{px}$ vertical. Figure 14c presents the trajectories of the markers calculated for the projection using the deformed (compensated) bitmaps. The maximum image drift was equal to $I_{max}=0.716\;\mathrm{px}$ horizontal and $J_{max}=0.576\;\mathrm{px}$. The positions of the markers printed on the artefact remained the same during the whole experiment (maximum shift was no higher than 0.168 px).

**Figure 15:**
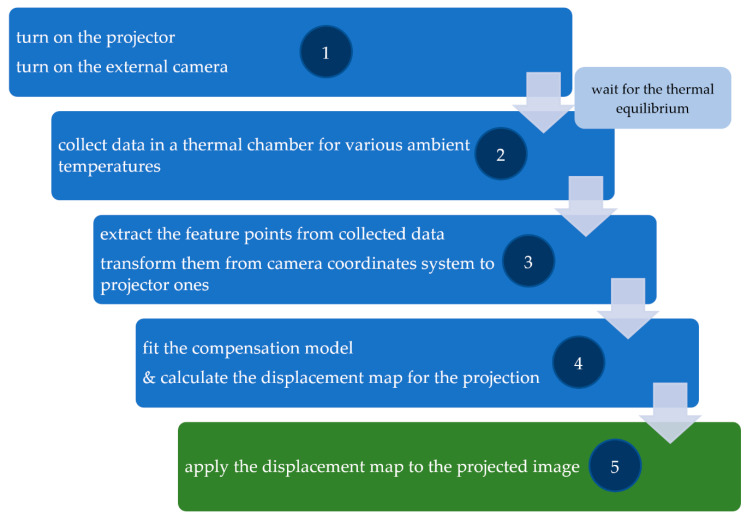
The compensation workflow.

The whole process of compensation is presented in Figure 15. The process starts after the projector and camera reaches its thermal equilibrium. Then the compensation data needs to be collected: the set of images with the pattern described in Section 3.2 (Figure 2d) in various ambient temperatures (Figure 10). The next thing is to extract the feature points (centers of projected markers) from collected data (Figure 11) and transform them into the projector coordinates system (Figure 1 and Figure 12). Then the compensation model can be calculated, and the displacement map for a particular temperature (from the temperature range determined in stage 2, Figure 15) can be estimated. The last thing to do is to apply the displacement map to the projected image in the corresponding ambient temperature.

### 4.4. Results

**Figure 16:**
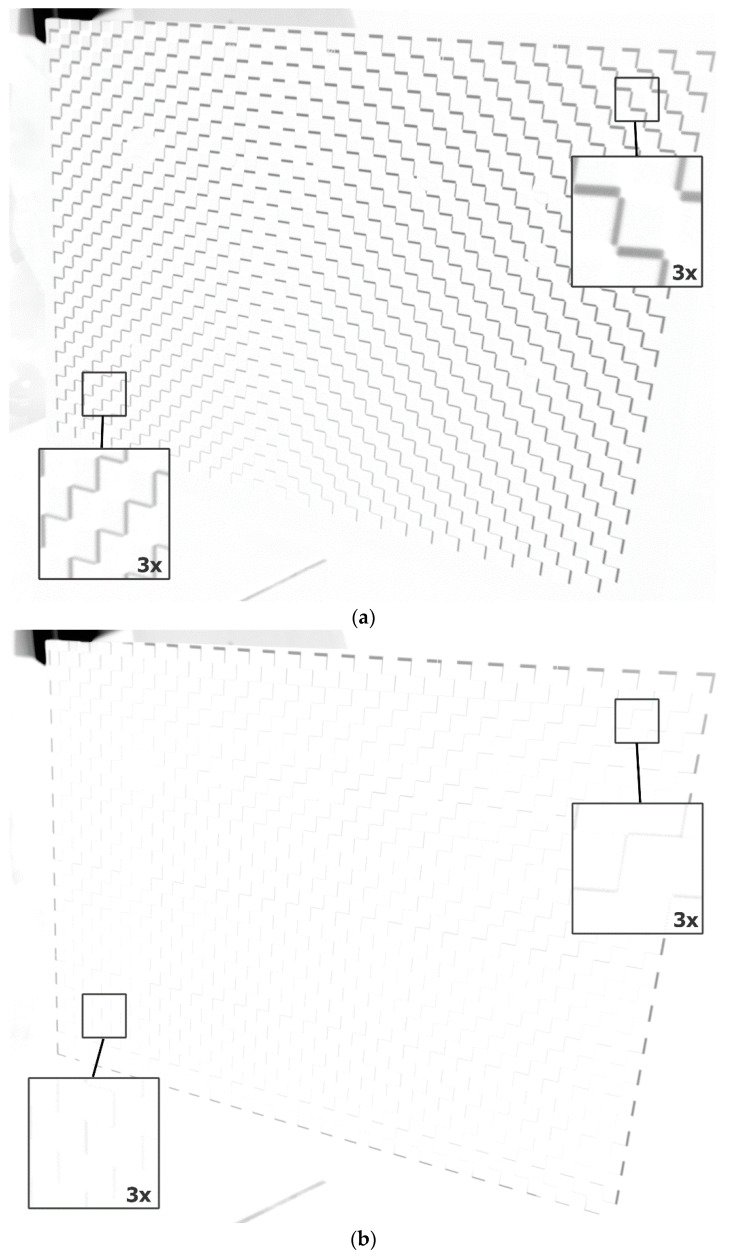
Qualitative demonstration of the effect of the calculated compensation model on the LightCrafter 4500 projector: (**a**) The presented image is the result of subtracting (and intensity inversion) two frames collected at reference temperature 42 ℃ and the temperature of 55 ℃ when projecting a black and white checkerboard pattern, without compensation of the projected image; and (**b**) the same frame subtraction, but with compensation of the projected image.

The presented results confirm that the calculated compensation model allows for a significant reduction in image deformation caused by changes in the external temperature of the projector. For the calculated coordinates of the markers, the deviation values achieved a 92% decrease for the horizontal direction and a 94% decrease for the vertical direction after compensation. Figure 16 shows the effects of the application of the compensation model more realistically and qualitatively. Figure 16a was created by subtracting the frame collected at the reference temperature and the frame collected at the temperature of 55 ℃ with the projection without compensation, where a black and white chessboard pattern (field width 25 px) was used as the projected image. In order to improve visibility, the intensity of the output image was additionally inverted. The edges associated with the image shift and its deformation caused by the temperature change of the projector can be observed. In the central and bottom parts of the projected image, the shifts are the smallest and propagate radially towards the image corners. This corresponds to the trend observed in the experiment described in Section 4.1.

Figure 16b shows the image resulting from the same subtraction of two frames captured at different temperatures (reference temperature 42 ℃ and 55 ℃) when using the proposed compensation model. It can be seen that the edges of the chessboard resulting from the subtraction of images are much less visible and that their thickness is the same over the entire area. Edges are clearly visible only at the edges of the projected image, which illustrates the difference between projection with and without compensation. The area where edges can be seen, however, is minimal, occupying a frame approximately 4–5 pixels thick at the edges of the image.

## 5. Discussion and Conclusions

This paper presented the results of research on the effects of temperature on the types of projectors which are commonly used in 3D SL scanners. The research was carried out using two models of projectors. It was shown that, during the projector warming-up process, as well as due to exposure to varying ambient temperatures, the projected image suffered significant deformation. These deformations reached values of up to several pixels in the projected image. Therefore, a model for compensation of the influence of changing temperature on projectors was also presented in this paper. Application of the developed compensation model led to a significant reduction in the thermal drift of the projected image. However, the presented compensation model also has disadvantages. One limitation is that it only works correctly in thermodynamically constant states. Thus, it cannot be used to compensate for the effect of temperature on the projected image during the warming-up process. Another fact that is crucial for the results of compensation is the repeatable behavior of the projector unit exposed to the varying thermal conditions. If the thermal drift of projected image changes in various experiments, the results of applying the proposed compensation model may not be as good.

Another limitation is the fact that, in order to develop a compensation model, it is necessary to have an appropriate test stand equipped with a thermal chamber. This can be a serious limitation, as access to a laboratory equipped with such an apparatus is relatively expensive and complicated. Proposed compensation model can be used in any raster projector, nevertheless, taking into account the complexity of the thermal issues, there is a need to calculate dedicated compensation model parameters for each projector unit.

The proposed method of compensation for the effect of changing temperature on projectors can be successfully used in 3D SL scanners. In order to integrate it with the existing 3D SL scanner, there is a need to equip the projector unit with the temperature sensor and implement the image deformation function in the software responsible for preparation of the measurement images. The calculations needed for the compensation are relatively simple, and it should not affect the speed of measurement of any 3D SL scanner. The presented verification experiments showed that, for the used projector, the thermal image drift could be reduced by over 90%. The proposed method does not require any interference in the construction of the projector; it is only necessary to equip the projector with an adequately positioned temperature sensor.
